# Multi-species plasmids and *K. pneumoniae* clonal spread contribute to *bla*_NDM_ outbreak across seven UK healthcare sites

**DOI:** 10.1093/jacamr/dlag216

**Published:** 2026-10-22

**Authors:** Caitlin Duggan, Charlotte Brookfield, David Lawrie, Victoria Owen, Timothy Neal, James Cruise, Alice J Fraser, Lewis Kelly, Fabrice E Graf, Daire Cantillon, Joseph M Lewis, Thomas Edwards, Eva Heinz

**Affiliations:** Department of Tropical Disease Biology, Liverpool School of Tropical Medicine, Liverpool, UK; Liverpool Clinical Laboratories, NHS University Hospitals of Liverpool Group, Liverpool, UK; Liverpool Clinical Laboratories, NHS University Hospitals of Liverpool Group, Liverpool, UK; Liverpool Clinical Laboratories, NHS University Hospitals of Liverpool Group, Liverpool, UK; Liverpool Clinical Laboratories, NHS University Hospitals of Liverpool Group, Liverpool, UK; Liverpool Clinical Laboratories, NHS University Hospitals of Liverpool Group, Liverpool, UK; Infection, Prevention and Control, Clatterbridge Cancer Centre NHS Foundation Trust, Liverpool, UK; Department of Tropical Disease Biology, Liverpool School of Tropical Medicine, Liverpool, UK; Department of Clinical Sciences, Liverpool School of Tropical Medicine, Liverpool, UK; Strathclyde Institute of Pharmacy and Biomedical Sciences, University of Strathclyde, Glasgow, UK; Department of Clinical Sciences, Liverpool School of Tropical Medicine, Liverpool, UK; Department of Tropical Disease Biology, Liverpool School of Tropical Medicine, Liverpool, UK; Department of Clinical Sciences, Liverpool School of Tropical Medicine, Liverpool, UK; Department of Clinical Infection, Microbiology and Immunology, University of Liverpool, Liverpool, UK; Tropical and Infectious Diseases Unit, NHS University Hospitals of Liverpool Group, Liverpool, UK; Department of Tropical Disease Biology, Liverpool School of Tropical Medicine, Liverpool, UK; Department of Clinical Sciences, Liverpool School of Tropical Medicine, Liverpool, UK; Strathclyde Institute of Pharmacy and Biomedical Sciences, University of Strathclyde, Glasgow, UK

## Abstract

**Objectives:**

The NDM carbapenemase enables Gram-negative bacteria to hydrolyse almost all β-lactam antibiotics. In 2023, a large increase of *bla*_NDM_ in clinical isolates was identified in a clinical microbiology laboratory serving hospitals and GP practices in Merseyside, UK, raising concerns of a larger outbreak.

**Methods:**

We used short-read WGS and bioinformatic analyses to identify the outbreak(s) and identify potential links by combining with anonymized, routinely collected clinical metadata. A subset of isolates were long-read sequenced to analyse resistance genes and plasmids at maximum resolution.

**Results:**

The outbreak included *Enterobacter hormaechei*, *Klebsiella pneumoniae*, *Escherichia coli*, *Citrobacter freundii*, *Citrobacter braakii* and *Enterobacter kobei* and 28 STs. *K. pneumoniae* ST101 represented the only major clonal expansion (18/21 *K. pneumoniae* isolates), and combination with metadata strongly suggested a multi-site hospital-associated outbreak. We identified four variants of *bla*_NDM_, and long-read sequencing indicated *bla*_NDM-1_ spread across species was driven by an IncHI2/IncHI2A plasmid, representing 82% of *bla*_NDM1_ isolates.

**Conclusions:**

This study captured an outbreak of *bla*_NDM_-positive Enterobacterales across eight healthcare settings and highlights the complex epidemiology of spread mediated by both expansion of successful lineages (*K. pneumoniae* ST101) and multi-species plasmids (IncHI2/IncHI2A). It also highlights the challenge of recognizing and tracing an outbreak linked to patients moving across different healthcare systems and the importance of linked surveillance systems.

## Introduction

In 2019, an estimated 1.27 million global deaths were attributed to bacterial antimicrobial resistance (AMR), with the highest number of deaths linked to *Escherichia coli* and *Klebsiella pneumoniae*.^1^ These bacteria, members of the Enterobacterales order, are particularly difficult to treat when they produce carbapenemase enzymes, which inactivate most β-lactam antimicrobials. They are associated with poor outcomes,^2^ and have been identified by the WHO as critical priority pathogens.^3^ In England, the incidence of carbapenemase-producing organisms is increasing rapidly, with 7438 cases of acquired carbapenemases reported to the UK Health Security Agency (UKHSA) in 2024, compared with 666 in 2021.^4^ An understanding of the reasons for this rise, and strategies to interrupt transmission, are urgently needed.

Treatment options are particularly limited for MBL carbapenemase enzymes,^5^ of which the NDM enzyme is most common in the UK,^4^ and worldwide.^6^ Originally described in 2009, the *bla*_NDM_ gene has by now spread across a large range of species with sequence data reporting up to 59 bacterial taxa,^7^ disseminated on plasmids often with a diverse bacterial host range. Transmission within hospitals is thought to be a key driver of dissemination to a vulnerable host population. Outbreak tracing of carbapenemase-producing Enterobacterales (CPEs) (including NDM producers) has historically focused on bacterial strain typing to infer transmission between patients or environmental sources,^8^ but newer sequencing techniques and advances in typing of mobile genetic elements (MGEs) like plasmids has allowed an assessment of the role of MGEs in driving multi-species spread of resistance genes.^9^

In this context, between 2022 and 2023, we noted a year-on-year increase in the number of inpatient samples positive for *bla*_NDM_ in the Liverpool Clinical Laboratories (LCL). We carried out a retrospective genomic-epidemiological investigation of *bla*_NDM_-encoding Enterobacterales to understand the drivers of this increase and the mechanisms of spread to guide prevention efforts.

## Methods

Full details of methods are available in the Supplementary methods (available as Supplementary data at *JAC-AMR* Online) and referenced here where appropriate.

### Study setting

The LCL serves seven adult hospitals (referred to here as Hospitals 1–6, and 8) and all GP practices throughout the city. These hospitals include two general acute hospitals with emergency departments and a number of specialty hospitals (Table S1), with complex care journeys for patients who may be transferred between sites to access specialty care, and are managed by several NHS trusts.

During the study period, high-risk hospital inpatients were screened for CPE colonization following UK guidelines,^10^ which includes testing rectal swabs by PCR (Xpert Carba-R assay), and as appropriate urine or wound samples (hereafter referred to as ‘screening samples’); clinical samples were also sent from hospital and GP sites when infection was suspected (hereafter referred to as ‘clinical samples’) and were processed according to standard methods (see Supplementary methods, ‘Clinical sample collection and processing’). Clinical samples that identified potential meropenem-resistant Gram-negative isolates by disc diffusion (zone size <28 mm) were tested for carbapenemase genes using the Xpert Carba-R assay (see Supplementary methods). Non-susceptibility to meropenem was confirmed or refuted by using a meropenem MIC Etest.

### Sample and metadata collection

We retrospectively identified all isolates in which *bla*_NDM_ was detected in the clinical laboratory from January 2023 to December 2023 using the laboratory information management system. There were 68 in total, and 63/68 of those could be used for further investigation and passed sample quality control, including 23 *Enterobacter* spp., 21 *K. pneumoniae*, 15 *E. coli* and four *Citrobacter* spp. The five samples that were unable to be used included four samples that showed no growth and one that was found to be a mixed culture of *E. coli* and *Enterobacter hormaechei* in short-read sequencing analysis.

Following HRA approval (IRAS ID 345990), we extracted anonymized linked patient metadata from electronic health records for these 63 isolates, including dates and times of hospital ward moves, though these ward move data were only available for Hospitals 1, 2, 3 and 5, which accounted for 60/63 isolates. We defined a strong epidemiological link between isolates as those cultured from different participants who had been on the same ward on the same day, and a weak link was defined as isolates cultured from samples collected from participants who had been on the same ward at any time (i.e. not necessarily on the same day). On the subset of participants who were sampled on the day of admission (Day 0), we explored hospital versus community acquisition: we defined hospital-acquired isolates as those which were subsequently cultured after a negative test on Day 0, hospital-associated as isolates present on Day 0 but cultured from participants who had been admitted to hospital in the last 6 months; and community acquired as isolates present on Day 0 with no recorded hospital admission in the last 6 months.

### WGS analyses

For full details of the sequencing and bioinformatic analysis see Supplementary methods. Briefly, all samples underwent short-read sequencing on an Illumina platform, and 24 were also subsequently sequenced on the Oxford Nanopore MinION platform using the Native Barcoding Kit 24 V14 (SQK-NBD114.24) (ONT, UK) and protocol. After quality control, either short-read only or hybrid genomes (for the isolates with long-read sequencing available) were assembled, and determinants of interest (AMR genes, capsule types, plasmid replicons) were determined. Plasmid assemblies were manually inspected, and hybrid-assembled plasmids and gene cassettes were compared for synteny and conservation (details in Supplementary methods).

### Phenotyping and plasmid conjugation

For *K. pneumoniae*, string tests were performed according to a previously published methodology;^11^ only two isolates encoded for yersiniabactin and yersiniabactin + aerobactin, and a partial *rmpA* gene, respectively (Table S2). Plasmid conjugation was attempted using an *E. coli* donor (NDM151) and *E. coli* MG1655 recipient using filter assays, and differential selection was used for colony counts and selection of transconjugants, which were confirmed by targeted PCRs (see Supplementary methods for full methods, and Table S3). The MIC of meropenem was identified for the donor, recipient and a transconjugant using MIC broth microdilution. The ATCC25922 *E. coli* strain was used as a quality control organism; endpoints were interpreted according to the EUCAST clinical breakpoints (v.15.0).

### Ethics statement

No identifiable data were analysed during this project. As an anonymized use of routinely collected data and following UK Health Research Agency (HRA) guidance the study did not require formal research ethics committee review. It was approved by the HRA (IRAS ID 345990).

## Results

### Sample and participant details

Between January and December 2023, a total of 68 isolates were identified as NDM-producing Enterobacterales in the clinical laboratory, of which 63 could be retrieved and passed quality control for further analysis. These 63 isolates were collected from 61 participants, with one environmental isolate (Table 1). Most isolates from humans were from colonization screens (56/63, 89%); 6 were screening samples (2 urine; 1 tissue; 2 wound swabs; 1 CSF). An additional environmental sample derived from an outbreak investigation at Hospital 2 (a shower trap) was added, resulting in a total of 63 samples. Over half of the isolates were from the two acute hospitals (Hospitals 1 and 5, 49% and 21%, respectively) and the specialist oncology hospital (Hospital 2, 17%); one isolate each was derived from Hospitals 4, 6 and 8, and one GP practice.

**Table 1.** Clinical details of sampled population^a^

Variable	Value
Participants’ age,^b^ y	
0–9	1/61 (2% [0–9%])
20–29	1/61 (2% [0–9%])
30–39	9/61 (15% [7–26%])
40–49	6/61 (10% [4–20%])
50–59	13/61 (21% [12–34%])
60–69	7/61 (11% [5–22%])
70–79	14/61 (23% [13–35%])
80–89	7/61 (11% [5–22%])
90–99	3/61 (5% [1–14%])
Location of sample isolation	
Hospital 1	31/63 (49% [36–62%])
Hospital 2	11/63 (17% [9–29%])
Hospital 3	4/63 (6% [2–15%])
Hospital 4	1/63 (2% [0–9%])
Hospital 5	13/63 (21% [11–33%])
Hospital 6	1/63 (2% [0–9%])
Community GP site 7	1/63 (2% [0–9%])
Hospital 8	1/63 (2% [0–9%])
Sample type	
Clinical	6/63 (10% [4–20%])
Environmental	1/63 (2% [0–9%])
Screening	56/63 (89% [78–95%])
Other clinical details	
Duration of hospital admission,^b^ median (IQR), d	23 (10–44)
Hospitalization within prior 6 months^b^	36/58 (62% [48–74%])
Previous negative CPE screen	35/61 (56% [43–69%])
Days since negative CPE screen, median (IQR)	136 (59–228)

Values in parentheses represent proportion i.e. n/N; squared brackets show 95% confidence interval.

^a^This included 61 participants, contributing 62 samples, and 1 environmental sample.

^b^Duration of hospital admission/prior hospitalization only available for Hospitals 1, 2, 3 and 5 representing 58/61 participants.

Age and gender data were available for all 61 participants (Table 1), and hospital and ward admission/discharge dates were available for all those admitted to Hospitals 1, 2, 3 and 5 (58/61). This is a highly healthcare-exposed cohort where hospitalization in the 6 months prior to CPE isolation was common (36/58, 62% of participants with available data) and median (IQR) duration of hospitalization for the episode where CPE was isolated was 23 days (10–44), a mean of 31.2 days compared with a mean duration of hospital stay for England of 8.3 days in 2022.^12^ The participants were admitted to 65 different areas in total, with a median of 3 (range 1–9) per participant. The most common admission was to critical care (H1_KH45), operating theatre (H1_CX45), an acute assessment unit (H1_RX30), and a surgical ward (H1_OV11), all within Hospital 1 (Figure S1). There were 16/58 (28%) participants admitted to multiple hospitals (Figure S2), 13/16 participants to two hospitals and 3/16 to three hospitals. Even with 58 patients, the network of ward movements within and between the four hospitals with available data formed a complex structure with clusters of highly connected wards [e.g. ICU (H1_KH45), theatres (H1_CX45), theatre recovery (H1_YV45), surgical ward (H1_YV45)] (Figure S3).

Participants were sampled a median (IQR) of 4 (2–14) days after admission; only 11 participants were sampled on the day of admission allowing an estimation of community versus hospital admission. Of these, 5/11 (45%) were not colonized on admission but subsequently became colonized and so fulfilled our definition of hospital-acquired; 5/11 (45%) were hospital-associated (colonized on admission but hospital admission within 6 months) and 1/11 (9%) was community-associated (colonized on admission and no recorded hospital admission). Strong epidemiological links were identified for 24/58 (41%) patients with location data (Figure 1b and d), consistent with possible transmission events, though they included isolates of different species (Figure 1e). These formed two large clusters (eight and six isolates, the larger including isolates from multiple hospitals), one smaller cluster (three isolates) and three isolate pairs (Figure 1e). Isolates with strong links were found in all hospitals with more than one isolate but were concentrated in Hospital 1, in particular ward H1_KH45 (Figure 1c and e), a critical care area where CPE screening is mandated for all patients.

**Figure 1.**
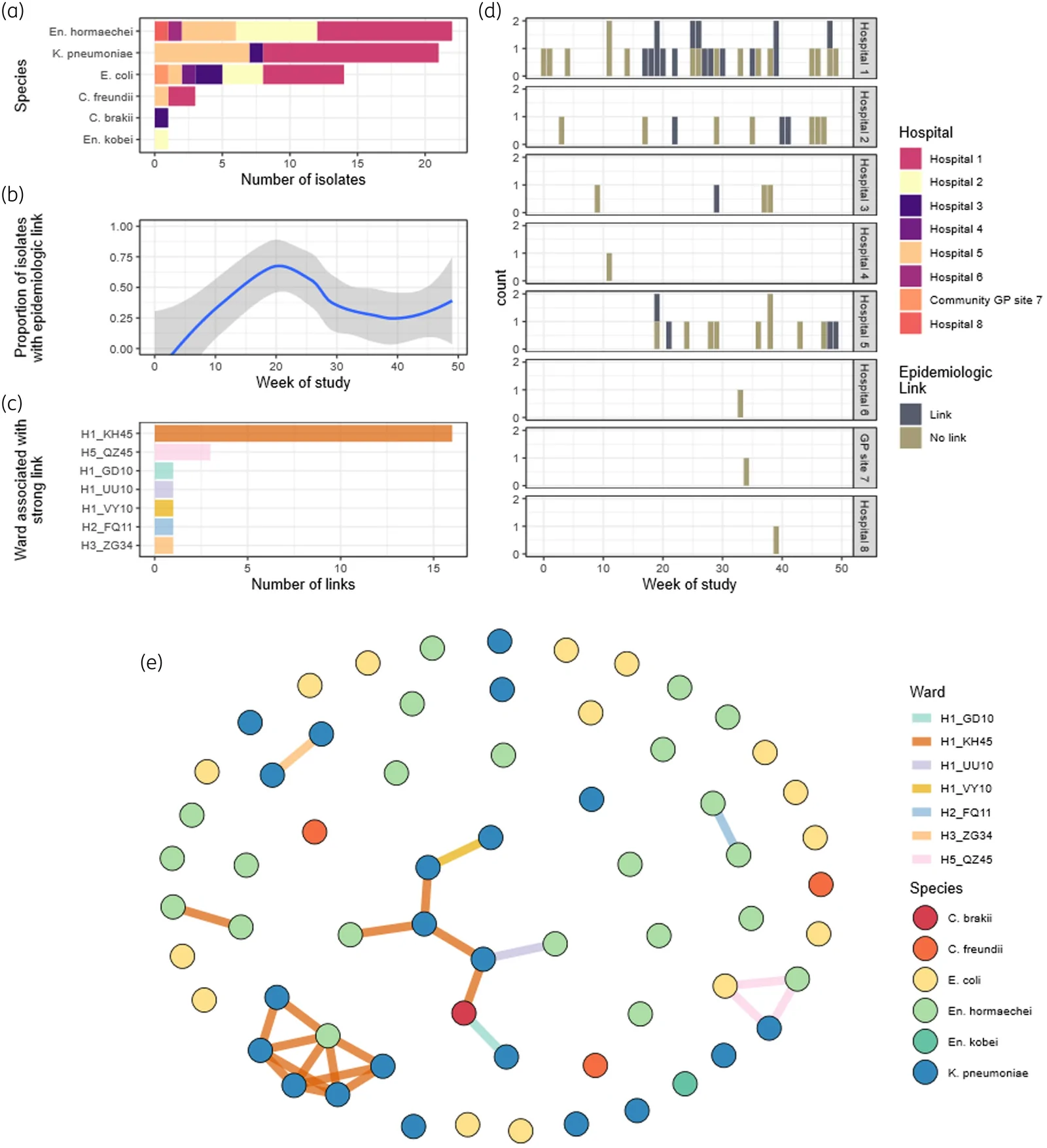
Epidemiological links within and between different species. (a) Species distribution of included isolates. (b) Proportion of isolates that had an epidemiological link to at least one other isolate. The grey shaded area on the graph refers to the 95% confidence level. (c) Hospital wards where strong epidemiological links occurred. (d) Isolates cultured per week stratified by site and colour based on at least one versus no epidemiological link to another sample. (e) Network map showing all isolates as nodes (coloured by species) and edges as strong epidemiological link (coloured by ward).

### Bacteria: STs and resistance determinants

Having identified possible epidemiological links, especially including distinct species, we proceeded to WGS and analysis of the 63 isolates to see if the genomic data were consistent with transmission events. This analysis confirmed six species (*E. hormaechei*, *n* = 22; *K. pneumoniae*, *n* = 21; *E. coli*, *n* = 15; *Enterobacter kobei*, *n* = 1; *Citrobacter freundii*, *n* = 3*; Citrobacter braakii*, *n* = 1) and 28 STs across all species (Figure 2). The most prevalent ST across the collection was *K. pneumoniae* ST101, representing 86% of all isolated *Klebsiella* in this study (18/21; Figure 2a and e). For the two other prevalent species, *E. hormaechei* and *E. coli*, the most prevalent STs represented only 18% and 27%, respectively (4/22 and 4/15). There were only two STs with more than one isolate within the *E. coli.* Across all species, 17 STs were represented by a single isolate; the majority of these (9/17) were *E. coli.* The one participant who contributed two samples was colonized with *E. coli* ST648 on two occasions, 2 days apart, with six core genome SNPs between them. For downstream analysis this was deduplicated by excluding the latter isolate, so all isolates were sampled from different participants.

**Figure 2.**
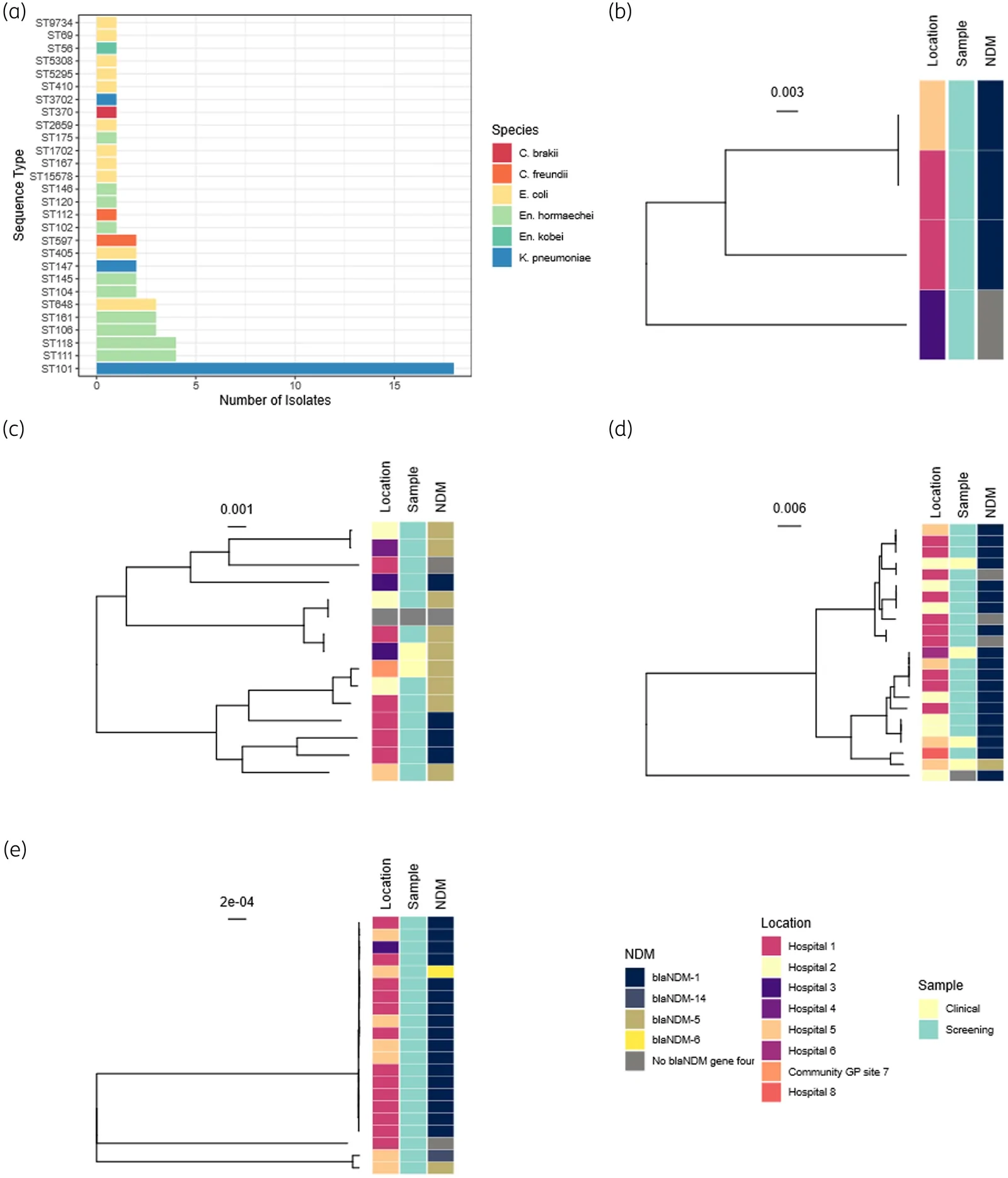
Population structure of the bacterial isolates. (a) STs for all included isolates. Phylogenetic trees for (b) *Citrobacter* spp., (c) *E. coli*, (d) *Enterobacter* spp. and (e) *Klebsiella pneumoniae*. Treescales show substitutions per base for a core gene pseudoalignment; trees are midpoint rooted.

### Multi-hospital clonal transmission driven by K. pneumoniae

We then used the WGS data to explore whether the strong epidemiological links identified could be consistent with transmission, first considering bacteria then MGEs. Of the 24 strong epidemiological links we found, 8/24 were of the same species and ST, and all of these involved *K. pneumoniae* ST101.

To assess whether the ST101 *K. pneumoniae* isolates represented a clonal outbreak, we performed a whole-genome SNP analysis, using a high-quality hybrid assembly of the earliest ST101 isolate as reference. Using rPinecone, we identified two sublineages comprised of 4 and 11 isolates each with fewer than 15 SNPs between isolates, which could be consistent with two putative transmission chains. Three more distantly related ST101 isolates did not belonging to a sublineage (Figure 3a–c). All ST101 *K. pneumoniae* isolates were colonization screening samples; all sublineage 1 isolates were sampled from Hospital 1 across a 5 month period with no strong and only one weak link in H1_KH45, the critical care unit for Hospital 1 (Figure 3d and e). Despite a lack of identified epidemiological links, all four patients carrying sublineage 1 were admitted to hospital in the previous 6 months: either Hospital 1 alone (two patients), Hospitals 1 and 5 (one patient) or Hospital 3 (one patient), possibly consistent with healthcare-associated (rather than community-associated) acquisition.

**Figure 3.**
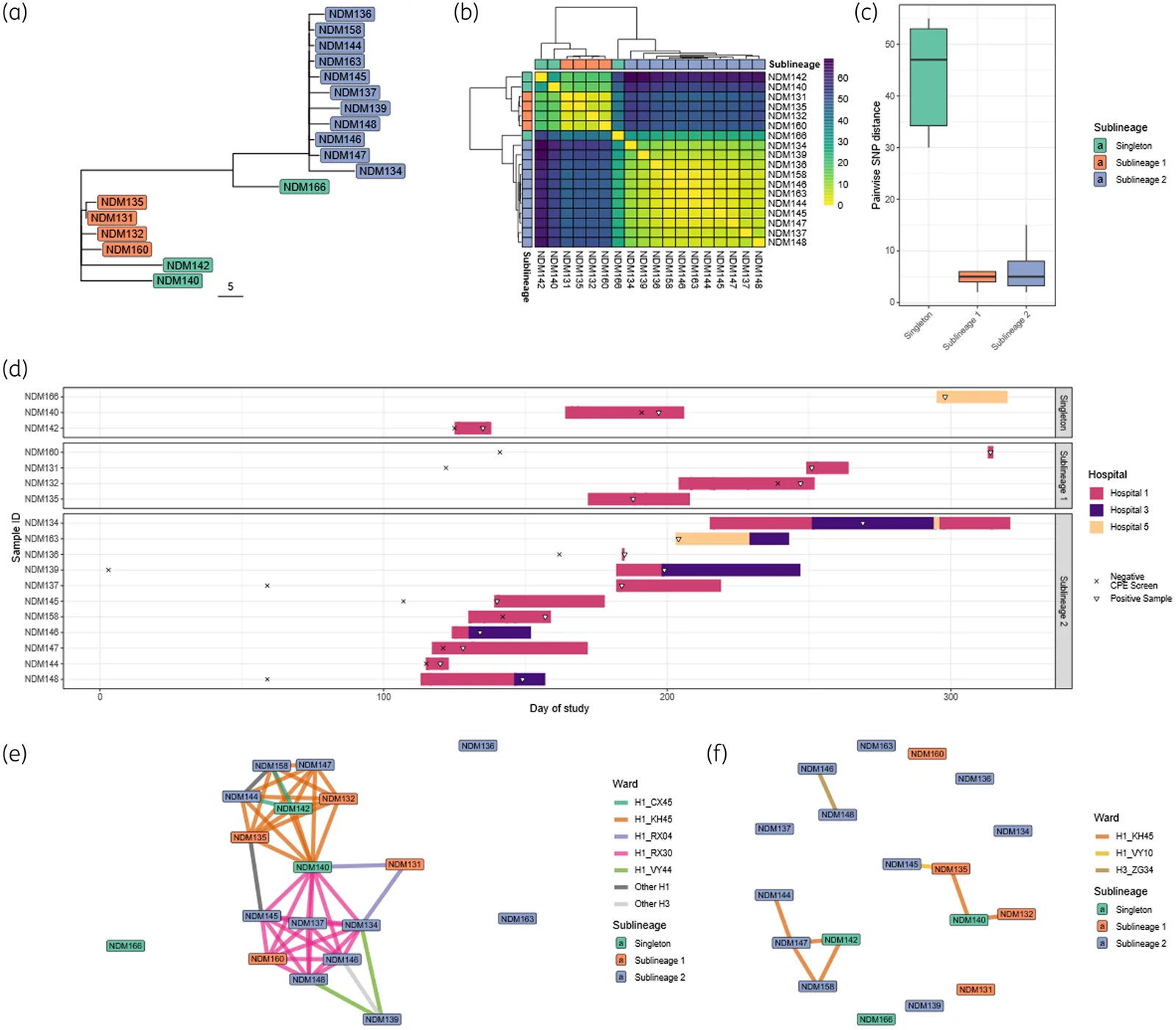
Exploring *K. pneumoniae* ST101 transmission. (a) Phylogenetic tree (midpoint rooted) derived from mapping reads to the earliest ST101 in the study as reference; tree scale here is absolute SNPs; tip colours show rPinecone sublineages. (b) Hierarchically clustered SNP-distance heatmap. (c) Pairwise SNP distances for the three sublineages. The central line of the boxplot is median, coloured boxes IQR and whiskers furthest point of distribution up to at most 1.5 * IQR. (d) Patient admission timeline showing duration of hospital admission for all participants (coloured bars indicating which hospital), times of prior negative CPE screening swab (crosses) and positive *K. pneumoniae* ST101 samples (white-filled shapes). (e,f) Weak (e) and strong (f) epidemiological links between isolates. Edges represent link (coloured by ward where link took place) and nodes are isolates, coloured by rPinecone lineage.

Sublineage 2 isolates were sampled from three of the hospitals within the study (Hospitals 1, 3 and 5), between May and October 2023. The first two sublineage 2 isolates were identified 8 days apart in H1_KH45, the critical care unit for Hospital 1; both patients were negative in earlier screens, on the same ward (Figure 3c), consistent with acquisition on that ward. A third sublineage 2 strain was isolated 29 days after the second from a patient in H1_KH45 who was similarly negative on admission screening, consistent with ongoing person-to-person transmission or via a shared source. Fourteen days after the second sublineage 2 ST101 isolate was identified at Hospital 1, another sublineage 2 ST101 isolate was identified at Hospital 5 (Figure 3c). No strong epidemiological link was identified, though this participant had been admitted to both Hospital 1 and Hospital 3 within the previous 6 months. One other strong epidemiological link for sublineage 2 (consistent with person-to-person transmission) was identified in Hospital 3. Both patients involved were transferred to Hospital 3 from Hospital 1, both had attended Hospital 1 within the previous 6 months, and both were admitted initially to Hospital 1 via ward H1_RX30, an acute medical unit, 11 days apart (representing a weak epidemiological link as per our definition, Figure 2e). A further three sublineage 2 isolates had been isolated from patients who passed through this ward, but not at the same time (i.e. weak epidemiological links as per our definition).

### Co-circulation of highly similar IncHI2 plasmids encoding for bla_NDM-1_ likely driving multi-species spread

Assessing acquired resistance genes for all WGS data detected 59 genes in total (Figure S4a, Table S4), with the majority conferring resistance to aminoglycosides and β-lactams (Figure S4a, b). The most common *bla*_NDM_ alleles were *bla*_NDM-1_ (43/63 isolates) and *bla*_NDM-5_ (12/63); and one each for *bla*_NDM-6_ and *bla*_NDM-14_. Additional carbapenemase genes of *bla*_IMP-1_, *bla*_VIM-1_, *bla*_VIM-1_ + *bla*_OXA-48-like_, and *bla*_OXA-48-like_ were also observed in *E. hormaechei*, *C. braakii* and *E. coli* in one isolate each (Table S4); all these isolates also encoded for a *bla*_NDM_. Although the isolates were originally selected for *bla*_NDM_ presence based on hospital diagnostics, no *bla*_NDM_ gene could be detected in WGS data of six isolates, most likely representing loss of the resistance gene or plasmid (Figure 4a).

**Figure 4.**
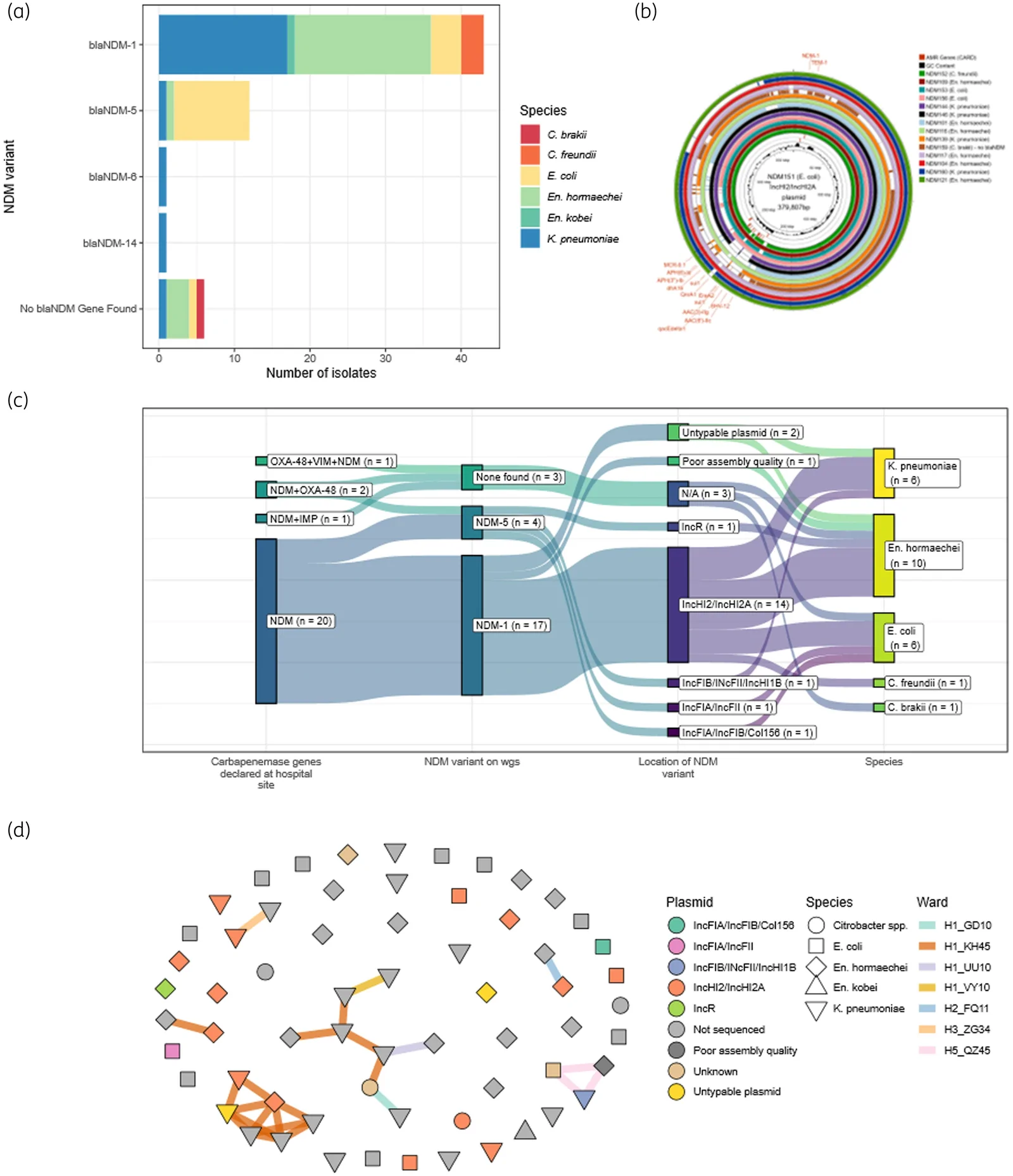
Genomic epidemiology of plasmids identified via hybrid assembly. (a) Distribution of *bla*_NDM_ alleles in all 63 isolates. (b) Proksee visualization of all 14 IncHI2/HI2A plasmids showing areas of sequence identity >80% compared with NDM151. (c) Sankey diagram showing association of NDM allele, plasmid and bacterial species. (d) Plasmid type (node colour) and bacterial species (node shape) overlaid on the network of strong epidemiological links (edges, coloured by ward of link). In the H1_KH45 ward–associated cluster of epidemiological links (bottom left) two different bacterial species carry the IncHI2/HI2A plasmid, consistent with a possible transmission event.

To resolve plasmid structures and genetic environments of *bla*_NDM_, we used long-read sequencing of 24 isolates, selected to represent the diversity of the collection in terms of plasmid replicon types (Table S1). The majority of isolates with *bla*_NDM-1_ (14/17, 82%) harboured the gene on an IncHI2/IncHI2A plasmid in a conserved gene cassette, flanked either side by IS*3000* transposons.^13^ Only two isolates encoded this gene on a different (untypeable) plasmid (Figure S5), and one had a poor quality assembly so a plasmid could not be identified. The 14 *bla*_NDM-1_ IncHI2/IncHI2A plasmids had a median size of 377 kbp (IQR, 338–385 kbp). In the first outbreak isolate carrying *bla*_NDM-1_ on IncHI2/IncHI2A (*E. coli*), this plasmid additionally harboured multiple other AMR genes, including *bla*_TEM-1_, *mcr-9* and *bla*_SHV-12_, which we used as reference in a comparison of closed IncHI2/2A sequences from our dataset (Figure 4b). These had high similarity of plasmid backbone with frequent insertions and deletions with a median BLASTn query cover of 96.5% (IQR, 91.25%–98.75%) to the reference IncHI2/IncHI2A plasmid in our dataset.

To explore the possibility that horizontal gene transfer could be responsible for *bla*_NDM_ transmission events in our hospitals, we looked for strong epidemiological links between isolates with the IncHI2/HI2A plasmid (Figure 4d). Only the 24 isolates subjected to long-read sequencing could contribute to this analysis; of these, 9/24 had a strong link to at least one other isolate. Of these, one isolate pair, including a sublineage 2 ST101 *K. pneumoniae* (NDM144) and an ST145 *E. hormaechei* (NDM145), both carried an IncHI2/HI2A plasmid and had a strong epidemiological link; they were present at the same time on H1_KH45, the critical care unit of Hospital 1.

To test whether the plasmid itself could be driving part of the outbreak we tested for ability to conjugate and demonstrated conjugation of the IncHI2/IncHI2A plasmid from an *E. coli* donor (NDM151) to the MG1655 *E. coli* strain recipient. We showed that this resulted in a meropenem-resistant phenotype (Figure S6, Tables S5 and S6). Using an *E. hormaechei* (NDM101) and *K. pneumoniae* (NDM160) donor with the MG1655 *E. coli* strain recipient was unsuccessful, likely because of lower conjugation rates for cross-species transfer.

In contrast, *bla*_NDM-5_ was detected on distinct plasmids in each long-read sequenced isolate, including IncR, IncFIA/IncFII multi-replicon, IncFIB(pNDM-Mar)/IncFII(29)/IncHI1B(pNDM-MAR) multi-replicon, and an IncFIA/IncFIB/Col156 multi-replicon plasmid (Figure 4c, Table S7). However, the flanking genes of *bla*_NDM-5_ included a conserved gene cassette of *ble*, *trpF* and *dsbD* that seems to translocate together with *bla*_NDM-5_. (Figure S7).

## Discussion

In 2023, an increase of NDM-producing bacteria was observed in the clinical microbiology laboratory serving the majority of people in Merseyside. Clinical investigation revealed that these isolates were largely from hospital inpatients and identified from colonization screening samples, but were spread across all the inpatient facilities in the city. Routine clinical metadata showed that the patients from whom these samples were collected had complex care journeys with frequent ward and hospital transfers, which took them under the care of different hospital trusts, clinicians, and infection prevention and control (IPC) teams. At the bacterial level, WGS showed that the spread of the *bla*_NDM_ gene was consistent with both clonal spread of *K. pneumoniae* ST101 and also multi-species spread of a successful IncHI2/IncHI2A plasmid encoding for the *bla*_NDM-1_ gene, in which conjugation was also demonstrated experimentally. There are several implications in these findings.

We identified evidence of patient-to-patient spread of *K. pneumoniae* ST101 in the critical care unit of Hospital 1 across several months, where CPE screening is performed weekly on all patients. SNP thresholds to identify likely transmission thresholds of bacteria have been widely debated,^14,15^ but the ST101 lineage 1 pairwise SNP distances (up to 6 SNPs) are consistent with person-to-person transmission or transmission from a shared source. In recent years, *K. pneumoniae* ST101 has emerged as a potential high-risk CPE clone in European countries, strongly associated with spread of CPE^16^ and carrying *bla*_KPC_ and *bla*_OXA_ carbapenemases as well as *bla*_NDM_, as in our study. The time scale of persistence of the strain is perhaps suggestive of an environmental reservoir, but we have no systematic environmental sampling to explore this hypothesis. Persistence of *Klebsiella* strains in the hospital environment, e.g. on shared equipment^17^ but also particularly in sinks,^18^ is well described as a cause of outbreaks. The increase in detection of NDMs locally resulted in an IPC investigation, and a number of interventions applied to reduce transmission (including education and reinforcement of standard IPC measures) after which no more links or *bla*_NDM_-positive isolates were identified within this ward.

In this study*, bla*_NDM1_ was very strongly associated with a multi-replicon IncHI2/IncHI2A plasmid. This was found in multiple bacterial species and on some occasions in patients who shared the same ward at the same time, consistent with transmission events at the level of the bacterial species and/or patient. The fall in the cost of long-read sequencing technologies makes it feasible to recover full genomic sequences of plasmids at scale, and plasmid-mediated multispecies CPE outbreaks are increasingly recognized;^19^ hence the role of plasmid transmission across different One Health compartments is being described.^9^ However, methods to compare plasmid similarity are still under development^20,21^ and the optimal method to infer plasmid transmission and incorporate tracking of plasmids into IPC outbreak investigations is unknown. This should be a priority for future research.

In contrast to *bla*_NDM-1_ encoded largely on IncHI2/IncHI2A, the *bla*_NDM-5_ gene was encoded by different IncF and IncR plasmids. Isolates carrying *bla*_NDM-5_ isolates were derived from three species in five hospitals and a community sample and so, in contrast to *bla*_NDM-1_ (and because of the diverse species and plasmid distribution) there were no strong epidemiological links with the same species or plasmid. It is possible therefore that transmission of these isolates could represent community acquisition, though without further sampling this must remain a hypothesis. Despite the different plasmid types, nearly identical flanking regions surrounded the *bla*_NDM-5_ including transposable elements, and consistent with a conserved transposable unit circulating in several species (IS*26*-*bla*_NDM-5_-*ble*-*trpF*-*dsbD*-IS*91*). Across England, in hospital settings the *bla*_NDM-5_ gene has been seen on IncFIA/IncFII multi-replicon plasmids in *E. coli* isolates, as in our study,^22^ consistent with national transmission.

At the health system level, our study highlights the importance of (and challenges in) tracking antimicrobial resistance or outbreaks across complex healthcare systems. Our hospitals in Liverpool are run by several different hospital trusts, with different data collection systems, different clinicians, managers and IPC teams. Coordinated action across this system is challenging but our data clearly demonstrate a multi-institution outbreak and highlight the importance of considering the healthcare system as a whole when considering action to tackle AMR. Sharing of data on AMR across organizations is key, and in our case was facilitated by a central microbiology facility.

This work had some limitations, mainly stemming from our use of routinely collected data. Screening for CPE was non-random, as per UKHSA guidance; the only areas where every patient was swabbed routinely was critical care; in other areas swabbing was based on admission risk factors or on Day 30 of admission, as per UKHSA guidance. This will introduce bias, and inflate the number of epidemiological links in critical care areas compared with other areas. Further information regarding the patient’s stay, such as the duration and locations visited within the hospital site, was unobtainable for some isolates in this study. Admission and ward-based data were not accessible for Hospitals 4, 6 and 8. This meant we were unable to identify any potential transmission events within these hospitals for any of our isolates. We lacked community surveillance data as CPE screening was targeted at those with recent healthcare exposure so our data do not describe the situation in the community. Though our data could be consistent with clonal spread of *K. pneumoniae* and multi-species spread of the IncHI2/IncHI2A plasmid, our sample size was small and we have no data on the regional epidemiology of NDM (or INcHI2) carriage to compare, which limits the inferences we can make.

### Conclusions

Overall, this work highlights how complex care journeys in modern healthcare systems can combine with the mobility of AMR genes and plasmids to drive spread of AMR. We observed *bla*_NDM_ disseminated by both clonal spread of *K. pneumoniae* ST101, and a successful multi-species IncHI2/IncHI2A plasmid. Patients frequently moving between wards and hospitals, including different NHS administrative sections, poses a logistical challenge to find the trade-off between data protection and rapid sharing of relevant information, like diagnostics of highly resistant isolates. To address the novel challenge posed by the AMR crisis it is necessary to consider the problem at the level of the health system and the patient, as well as bacterial species, plasmids and gene cassettes, all of which are essential to resolve in order to accurately track a complex multi-species multi-hospital outbreak as in our report.

## Supplementary Material

dlag216_Supplementary_Data

## Data Availability

All sequence data are available under bioproject PRJEB89345; a detailed overview of accessions is given in Table S1. All underlying data and methods are provided in the publication or as supplementary information.

## Supplementary data

Supplementary methods, Figures S1 to S7, and Tables S1 to S7 are available as Supplementary data at *JAC-AMR* Online.
